# Supplementary material for: The Plot Thickens: Haploid and Triploid-Like Thalli, Hybridization, and Biased Mating Type Ratios in Letharia
Source: Front Fungal Biol. 2021 May 26;2:656386. doi: 10.3389/ffunb.2021.656386 (PMC10512270; doi:10.3389/ffunb.2021.656386)
Supplement: Supplementary file 2 [file Data_Sheet_2.DOCX]

Supplementary Material

# Supplementary Methods and Results

## Identification of *Letharia* specimens

We used the ITS marker as a barcode, together with thallus morphology (sorediate vs. apotheciate) to assign *Letharia* thalli to the previously described species (Kroken and Taylor 2001a; Altermann 2009; Altermann et al. 2016). In addition, we found 18 unique ITS variants (**Table S1**). The specimens with unique ITS variants were putatively assigned to previously known lineages based on thallus morphology and ML analyses of the ITS and marked with “cf.” in **Figure S1**. The ITS alignment, including variants unique to this study, and previously published variants, was 555 bp long with 69 variable sites. The ITS trees are in concordance with previously published results (Kroken and Taylor 2001a; Altermann et al. 2014; Altermann et al. 2016). Two Italian specimens had a 100% identical match to previously published ITS variants extracted from *L. gracilis*, *L. vulpina* and *L. lupina* (Altermann 2009). These specimens were putatively assigned to *L. vulpina* based on the identity of the symbiotic alga from the thallus (ITS sequence, data not shown), following Altermann (2009) and Altermann et al. (2016). We had eight specimens putatively assigned to *L. gracilis* in our dataset (morphology-based identification), but the ITS variants for these did not match the previously published *L. gracilis* variants but instead those of *L. lupina* or *L. vulpina* (**Table S1**). These specimens were designated *L. cf. gracilis* and their species identity cannot be resolved with current data.

## Training of *ab initio* gene predictors for *Letharia* genomes

The program SNAP was trained a priori with the *L. lupina* transcripts produced by Trinity, following the instructions in the SNAP’s README and Campbell et al. (2014). The MAKER pipeline v. 2.31.8 (Holt and Yandell 2011) was run to infer gene predictions directly from the Trinity transcripts (*est2genome=1*). The resulting GFF files were transformed into the non-standard format ZFF using the script *maker2zff*. All models with warnings or errors reported by the program fathom where excluded. The program forge was used to estimate parameters for the script hmm-assembler.pl. The resulting HMM training files were used to run MAKER once more, this time with *est2genome=0*. SNAP was retrained as above using this new MAKER annotation. The HMM training files of the retraining process were used for annotation in downstream analyses. Augustus was trained using the protein sequences of the gene models produced by running SNAP on the SPAdes assembly of *L. lupina*. We used the script *autoAug.pl* (part of Augustus distribution) to do the training with *--maxIntronLen=1000*. Finally, GeneMark was self-trained using the script *gmes_petap.pl* and the flags: -*-fungal --max_intron 3000 --min_gene_prediction 120*. All scripts used and the training files are available at<https://github.com/johannessonlab/Letharia>.

## Additional results of the MAT region annotation

The metatranscriptomic data supported the presence of a small ORF (herein referred to as *lorf*, from *Letharia* open reading frame) between *APN2* and *MAT1-2-1* in *L. columbiana*, flanking the beginning of the idiomorphic region (**Figure 2** and **Figure S8**). In the other species, however, *lorf* displays relatively low levels of expression (see **Figure S8-S11**), which may be due to differences in biological conditions during the time of collection. In the *L. lupina* metatranscriptome, TransDecoder detected *lorf* within the transcripts, suggesting activity in this species. Nevertheless, an alignment of all samples (including sequences acquired from PCR) revealed a potentially on-going process of pseudogenization in the entire genus. All the *lorf* sequences present in samples of the MAT1-1 idiomorph have two potential start codons (AUG), while some *lorf* sequences next to a MAT1-2 idiomorph have either one start codon, or none. Some samples also have frame-shift mutations. Noteworthy, *lorf* is located at the border between the idiomorph and the flanking region (**Figure 2**) and shows a slight divergence (~96% of similarity) between MAT1-2 (*L. columbiana*) and MAT1-1 (*L. lupina*, *L. vulpina*, and *L. ‘rugosa’*).

In addition, the *Letharia* transcript models for the *MAT1-1-1* gene were consistently predicted in the opposite sense, based on homology with other fungal species and the *ab initio* gene predictors. This antisense transcript (**Figure 2A**) was present in all *Letharia* species, including the transcriptome of the pure culture of *L. lupina*. We recovered the transcript in the canonical sense for *MAT1-1-1* along with antisense transcripts only for *L. ‘rugosa’* (**Figure 2**; see also **Figure S8-S11**).

## Nomenclature of auxiliary MAT genes

The nomenclature for mating-type genes as suggested by Turgeon and Yoder (2000) is used for most fungal groups. Under this system, whenever a new gene is discovered, with no detectable homology to any other, an incremental number is assigned to it following certain guidelines (Turgeon and Yoder 2000). Unfortunately, there is no centralized database associated with all published auxiliary MAT genes, which has led to a number of inconsistencies and overlaps with gene names (Wilken et al. 2017). For example, genes homologous to the auxiliary gene in *Letharia* have previously been named *MAT1-1-4*, *MAT1-1-7*, or *MAT1-1-9* (**Figure 3**, left)*,* depending on the author (Wilken et al. 2017). As discussed by Wilken et al., this gene is not related to the *MAT1-1-4* present in the Leotiomycete *Pyrenopeziza brassicae*, so this name is not appropriate. However, we disagree with Wilken et al. (2017) in giving a separate name for *MAT1-1-*7 and *MAT1-1-9* (and perhaps even the *MAT1-1-8* gene present in the Dothidiomycete *Shaeropsis sapinea*) on the basis of low pairwise identity, as the high divergence between these genes is coherent with the large phylogenetic distance between these fungi. Hence, we refer to the *Letharia* gene as *MAT1-1-7*, which is the smallest number available. Incidentally, Armaleo et al. (2019) also used this name for the ortholog in *Cladonia*.

Since the auxiliary gene in the MAT1-2 idiomorph of *Letharia* has no detected homologs outside Lecanoromycetes, a new number should be assigned. The highest number in use that we could identify is *MAT1-2-12*. However, two independent studies used the name *MAT1-2-12* for the genes in unrelated taxa: *Teratosphaeria* (Aylward et al. 2019) and *Calonectria* (Li et al. 2020). We confirmed that these genes are not homologous to each other, or to the gene in *Letharia*, using BLASTp searches. Hence, the correct name for the *Letharia* auxiliary gene is *MAT1-2-14* and the genes in *Calonectria* (Li et al. 2020) should be called *MAT1-2-13* based on the date of publication.

# Supplementary Figures and Tables

## Supplementary Tables

**Table S1.** *Letharia* specimens used in this study and detailed information on their collection sites, obtained MAT idiomorphs and data repository. The taxon identification is based on the ITS variants and thallus morphology. The ITS variants are named with the codes used by Kroken and Taylor (2001a) and Altermann et al. (2014).

(see provided Excel file)

**Table S2.** Assembly statistics of the draft assemblies used in this study.

^†^ All metrics are computed for contigs > 500 bp.

**Table S3.** Primers used for the PCR amplification and sequencing of the MAT locus in *Letharia*. The primers with no annealing temperature were used only for sequencing. The ITS primers were used to amplify the ITS region for all studied specimens (Tuovinen et al., 2019).

†Used for PCR screen of the mating type ratios in the populations

## Supplementary Figures

**Supplementary Figure 1.** Unrooted maximum likelihood tree of ITS with all obtained variants from this study (marked with U, Sw or It) and previously published ones, the code referring to the sequences from Kroken & Taylor (2001a), Alterman et al. (2014), Alterman et al. (2016) or NCBI accession number. The unique variants obtained in this study are marked red. The Italian variant marked with * is assigned to *L. vulpina* based on algal ITS. Branch lengths are proportional to the scale bar (nucleotide substitutions per site).

**Supplementary Figure 2.** Maximum likelihood phylogeny of the *Letharia* genus produced from 11 concatenated markers. Colours highlight specimens with our Illumina data and other sequences are from previous studies. Numbers above branches represent bootstrap support values. Branch lengths are proportional to the scale bar (nucleotide substitutions per site). The tree was rooted arbitrarily with the highly supported clade containing the metagenome of *L. columbiana*. MG: metagenome.

**Supplementary Figure 3.** Dotplot of MUMmer alignments between the assemblies of the McKenzie *L. lupina* metagenome and our *L. lupina* pure culture. Scaffold names of the pure culture assembly are omitted for clarity. Blue indicates alignments in the same sense, while red marks inversions. The Contig 1 of the McKenzie *L. lupina* assembly is excluded since alignments are too short and scarce to be visible.

**Supplementary Figure 4.** Dotplot of MUMmer alignments between the Contig 1 of the McKenzie *L. lupina* metagenome and our *L. lupina* pure-culture assembly. Blue indicates alignments in the same sense, while red marks inversions.

**Supplementary Figure 5.** Repeat content proportions of the contigs in the McKenzie *L. lupina* metagenome assembly.

**Supplementary Figure 6.** Coverage (in total number of base pairs) of the top five most abundant repetitive elements in the Contig 1 from the McKenzie *L. lupina* metagenome across the biggest scaffolds of the *L. lupina* pure culture assembly.

**Supplementary Figure 7.** Coverage (in total number of base pairs) of the top five most abundant repetitive elements in the Contig 1 across all the other contigs of the McKenzie *L. lupina* metagenome assembly.

**Supplementary Figure 8.** Annotation of the MAT locus of *Letharia columbiana*. The main sources of evidence for annotation are shown. These include the *ab initio* gene models (Augustus, GeneMark and SNAP), transcript models (Cufflinks), and ORFs from transcripts (TransDecoder). The final models are presented in black. At the bottom, we present the depth of coverage per site from the metatranscriptome.

**Supplementary Figure 9.** Annotation of the MAT locus of *Letharia lupina*. The main sources of evidence for annotation are shown. These include the *ab initio* gene models (Augustus, GeneMark and SNAP), transcript models (Cufflinks), and ORFs from transcripts (TransDecoder). The final models are presented in black. Note that the transcriptomic models and ORFs for both the pure culture and the lichen thallus (metagenome) are shown. The depth of coverage at the bottom is only for the pure culture.

**Supplementary Figure 10.** Annotation of the MAT locus of *Letharia ‘rugosa’*. The main sources of evidence for annotation are shown. These include the *ab initio* gene models (Augustus, GeneMark and SNAP), transcript models (Cufflinks), and ORFs from transcripts (TransDecoder). The final models are presented in black. At the bottom, we present the depth of coverage per site from the metatranscriptome.

**Supplementary Figure 11.** Annotation of the MAT locus of *Letharia vulpina*. The main sources of evidence for annotation are shown. These include the *ab initio* gene models (Augustus, GeneMark and SNAP), transcript models (Cufflinks), and ORFs from transcripts (TransDecoder). The final models are presented in black. At the bottom, we present the depth of coverage per site from the metatranscriptome.

**Supplementary Figure 12.** Maximum likelihood trees of the alignment of (**A**) MAT1-1 (encompassing the *MAT1-1-7* and *MAT1-1-1* genes along with their intergenic region), (**B**) MAT1-2 (*MAT1-2-1* and *MAT1-2-14* with their intergenic region), and the fungal barcode ITS (**C**). Bootstrap values are shown above the branches. Branches with bootstrap values above 70% are thickened. Colours highlight the four main taxa under study: *L. columbiana* in purple, *L. ‘rugosa’* in orange, *L. vulpina* in blue and *L. lupina* in green. Samples with genomic or metagenomic data are in bold. Trees were rooted arbitrarily with *L. columbiana.* Branch lengths are proportional to the scale bar (nucleotide substitutions per site). Notice that previous studies have found extensive conflict between markers (Kroken and Taylor 2001a; Altermann et al. 2014; 2016) and so the ITS topology might not reflect the species tree.

**Supplementary Figure 13.** Depth of coverage distribution across different types of sites in the *L. lupina* metagenome. Using the *L. lupina* pure culture as reference, all variable sites were classified as “lupina” if they are fixed (not variable) and identical to the reference, as “other” if they are fixed for an alternative allele, or polymorphic if they have two alleles at a given site. (**A**) The majority of sites fall into the “lupina” category, but this category also includes many sites that were considered variable because one or more of the other species (*L. columbiana*, *L. vulpina* and *L. ‘rugosa’*) were different from the reference at those given positions. In other words, invariable sites across samples get filtered out. To make their coverage easier to compare, the sites fixed for the “lupina” allele and those that are polymorphic were subsampled to the same number of sites as the “other” allele in (**B**). Red dots represent the median of each coverage distribution.


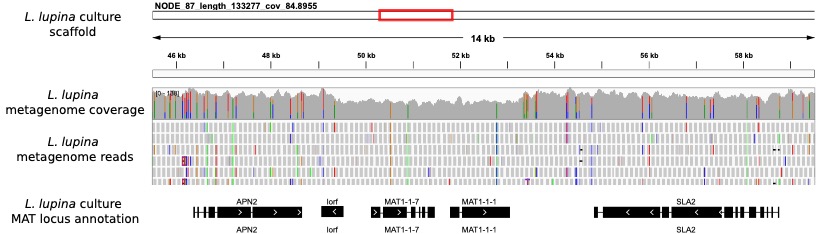


**Supplementary Figure 14.** Depth of coverage of the *L. lupina* metagenome reads mapped along the MAT idiomorph as displayed by the Integrative Genomics Viewer (IGV) program. The pure culture assembly (MAT1-1) was used as reference. The coverage within the idiomorph drops to approximately two thirds of the normal coverage, as expected for a 2:1 proportion of the MAT1-1 and MAT1-2 subgenomes within the *L. lupina* metagenome data. Colored columns along the coverage track represent the polymorphic sites and their frequencies.

**Supplementary Figure 15.** Allele frequencies and ancestry of the *L. lupina* metagenome variants around selected scaffolds. The *L. lupina* pure culture was used as reference to classify each allele as either “lupina” (reference) or “other” (alternative). Points represent the raw allele frequency at each site, while the solid lines connect the median allele frequency of non-overlapping 2.5 kb-long windows. Sites and windows overlapping with repetitive elements were discarded (missing data). Below the allele frequencies of each scaffold, two horizontal bands represent the inferred sister of the “other” (upper band) and “lupina” (lower band) components across the contig, inferred from genealogies of non-overlapping windows of 50 SNPs each. Genealogies were rooted with *L. columbiana*.
